# ChIP-Seq and RNA-Seq Analyses Identify Components of the Wnt and Fgf Signaling Pathways as Prep1 Target Genes in Mouse Embryonic Stem Cells

**DOI:** 10.1371/journal.pone.0122518

**Published:** 2015-04-13

**Authors:** Audrey Laurent, Manuela Calabrese, Hans-Jörg Warnatz, Marie-Laure Yaspo, Vsevolod Tkachuk, Miguel Torres, Francesco Blasi, Dmitry Penkov

**Affiliations:** 1 IFOM (FIRC Institute of Molecular Oncology), IFOM-IEO-Campus, Milan, Italy; 2 Department of Vertebrate Genomics, Max Planck Institute for Molecular Genetics, Berlin, Germany; 3 Faculty of Basic Medicine, Lomonosov Moscow State University, Moscow, Russia; 4 Department of Cardiovascular Development and Repair, Centro Nacional de Investigaciones Cardiovasculares (CNIC), Madrid, Spain; 5 Department of Experimental Cardiology, Russian Cardiology Research and Production Complex, Moscow, Russia; Instituto Gulbenkian de Ciência, PORTUGAL

## Abstract

The Prep1 (Pknox1) homeodomain transcription factor is essential at multiple stages of embryo development. In the E11.5 embryo trunk, we previously estimated that Prep1 binds about 3,300 genomic sites at a highly specific decameric consensus sequence, mainly governing basal cellular functions. We now show that in embryonic stem (ES) cells Prep1 binding pattern only partly overlaps that of the embryo trunk, with about 2,000 novel sites. Moreover, in ES cells Prep1 still binds mostly to promoters, as in total embryo trunk but, among the peaks bound exclusively in ES cells, the percentage of enhancers was three-fold higher. RNA-seq identifies about 1800 genes down-regulated in *Prep1* ^-/-^ ES cells which belong to gene ontology categories not enriched in the E11.5 *Prep1^i/i^* differentiated embryo, including in particular essential components of the Wnt and Fgf pathways. These data agree with aberrant Wnt and Fgf expression levels in the *Prep1* ^-/-^ ES cells with a deficient embryoid bodies (EBs) formation and differentiation. Re-establishment of the Prep1 level rescues the phenotype.

## Introduction

Prep1 (Pknox1) homeodomain transcription factor belongs to the TALE (Three Amino acids Loop Extension) family of proteins that includes also Prep2, Pbx1-4 and Meis1-3 [1, 2]. Prep1 dimerization with Pbx proteins through the amino-terminal domain provides DNA-binding activity to both proteins. In turn, the dimers can bind anterior Hox proteins through the Pbx homeodomain, forming functional trimers with higher DNA affinity and selectivity [3–8].

*Prep1* ^-/-^ embryos die before gastrulation because the epiblast undergoes p53-dependent apoptosis around E6.25 [9]. On the other hand, hypomorphic *Prep1* ^*i/i*^ embryos (expressing only 2% of the normal *Prep1* mRNA level) die mostly at E17.5 with an hematopoietic phenotype [10]. Double *Prep1* ^*-/i*^ heterozygotes display an intermediate phenotype dying at E12.5 with major cranial and brain defects [11]. Thus Prep1 is essential in at least three different stages of development: pre-gastrulation (0% expression), brain-cranium formation (1%) and hematopoiesis (2%). So the expression of different, even if low, levels of *Prep1* can have a major impact on the embryo phenotype.

In the adult, Prep1 behaves as a tumor suppressor since the few surviving hypomorphic *Prep1* ^*i/I*^ and the apparently normal *Prep1* ^*+/i*^ heterozygous mice develop a variety of tumors with age. Likewise, Prep1 haploinsufficiency accelerates Myc-driven lymphomagenesis [12, 13]. The underlying mechanism appears to be DNA damage since *Prep1* down-regulation acutely causes an accumulation of double-strand DNA breaks and chromosomal aberrations [14]. Therefore, *Prep1* exerts multiple functions in different cells during mouse embryo development and in the adult. Moreover, *Prep1* has an essential role in at least some stem cells, since the function and self renewal of hematopoietic stem cells is deficient in *Prep1* ^*i/i*^ embryos [15, 16]. Moreover, *Prep1* ^-/-^ blastocysts produce fewer and deficient ES cells [9].

Despite its almost ubiquitous expression pattern [17], the different *Prep1* phenotypes in different cells suggest that the identification of target genes may help discriminating between its different functions. On the basis of the above considerations, one would expect different genes to be targeted in different cells. We have previously identified by ChIP-seq the Prep1 target genes in the whole E11.5 embryo trunk [18] which represents a mixed population of embryonic progenitors and differentiated cells. Prep1 binds to a specific decameric DNA sequence at over three thousand genes, with great preference for promoters and mostly in the form of a Pbx1 heterodimer. Despite the genetic relevance of *Prep1* in embryonic development, Gene Ontology (GO) analysis showed that in the cells of the embryo trunk Prep1-bound genes are enriched particularly in categories involved much more in basic cellular functions than in developmental regulation [18].

Embryonic stem (ES) cells are pluripotent stem cells that derive from the blastocyst and are able, when re-implanted in the blastocyst, to reproduce an entire embryo. Moreover, they can be differentiated *in vitro* into a variety of tissues [19]. Prep1 and Pbx1 proteins are expressed in ES cells and the Pbx1 level is regulated upon differentiation [9].

In ES cells, because of the very low level of Meis1, the Prep1-Pbx complexes can bind DNA in the absence of the potentially antagonizing Meis1-Pbx1 dimers. Identifying target genes in ES cells might give information on Prep1 functions which might have escaped in the whole E11.5 embryo trunk analysis because of the major contribution of differentiated cells and of the presence of different progenitors.

We have identified Prep1 target genes in ES cells combining DNA sequencing and chromatin immunoprecipitation (ChIP-seq), and compared globally the relative gene expression level in wild type (WT) v. *Prep1* ^-/-^ ES cells (RNA-seq). While confirming the general rules of binding of Prep1 to DNA drawn from the embryo analysis [18], we have also uncovered major specific differences. First, in ES cells Prep1 targets a gene population that overlaps only by one third with that of the embryo trunk, thus enlarging the spectrum of the Prep1 binding sites. In particular in ES cells Prep1 targets several components of the Fgf and Wnt signaling pathways, which were not apparent in the embryo study. The control by Prep1 of Fgf and Wnt pathways is evidentiated by a deficient expression of some of their members in *Prep1* ^-/-^ ES cells upon *in vitro* differentiation.

Since Prep1 binds DNA preferentially as a Pbx dimer, and since Pbx can form dimers also with other transcription factors of the same TALE family, the phenotype of the *Prep1* deletion may be due not only to the absence of Prep1 (loss of function), but also to a relative increase of antagonistic dimers (gain of function). Instead, we show that in *Prep1* ^-/-^ ES cells the level of Pbx1 and Pbx2 is strongly decreased in *Prep1* ^-/-^ ES cells. Finally, re-establishment of the Prep1 level in *Prep1* ^-/-^ ES cells rescues the deficiency in embryoid bodies formation at the cellular and at least in part at the molecular level.

## Results

### Prep1 ChIP-seq in mouse ES cells identifies novel target genes

The original ChIP-seq and RNA-seq data of this experiment have been deposited in GEO and can be accessed with the GSE63282 accession number.

We performed a ChIP-seq study of wild type ES cells using an anti-Prep1 antibody (see Methods). The fragmented chromatin (average size 200 bp) was immunoprecipitated and the DNA deep-sequenced alongside with the non immunoprecipitated input. Using a threshold *p*-value <10^-6^ and an FDR (false discovery rate) <20% in the immunoprecipitation v. input comparison, we identified 3902 peaks bound by Prep1 (S1 Table) and mapped them with respect to transcriptional start sites (TSS) (Table 1). Of all peaks, 1271 were associated to a TSS (from -500 to +100). The TSS-distal peaks were classified as intragenic, close intergenic (<20 Kb from TSS) and far intergenic (>20 Kb) (Table 1). Cross-analysis with published data from ES cells [20–22] established that 1152 of the TSS-proximal peaks had histones modification marks (H3K4Me3^+^ and RNA-Pol-II^+^) identifying active promoters. The same promoter features were shared also by some TSS-distal peaks: 437 intragenic, 252 close intergenic and 169 far intergenic (Table 1), bringing the total of H3K4Me3^+^ and RNA-Pol-II^+^ peaks (i.e. promoters) to 2010.

**Table 1:** Overall view of the Prep1 ChIP-seq Peaks in wild type ES cells^#^.

Peaks	n
Total	3902
TSS-proximal peaks *	1271
TSS-proximal Promoters (H3K4Me3^+^, RNA-Pol-II^+^)^§^	1149
TSS-distal Enhancers (H3K4Me1^+^, H3K4Me3^-^)^§^ °	395
TSS-distal peaks: intragenic (total/promoter marks positive)	1238/432
TSS-distal peaks: close intergenic (<20Kb) (total/promoter marks positive)	608/252
TSS-distal peaks: far intergenic (>20Kb) (total/promoter marks positive)	785/169

^#^ We only considered ChIP-seq peaks with p-value <10^-6^ (based on the comparison between immunoprecipitated and non immunoprecipitated input samples.

* Located between -500 and +100 bps from a transcriptional start site.

^§^ The presence of RNA polymerase II and of the indicated histones modification was taken from [20, 21].

° Two additional peaks with the same enhancer marks are present among the TSS-proximal peaks.

Likewise [22], 393 non TSS-associated Prep1-bound peaks had histone modifications (H3K4Me1^+^ and H3K4Me3^-^) identifying TSS-distal enhancers (Table 1). Only two peaks with these properties were detected in proximity of a TSS (see Table 1 legend). Overall, the data confirm in ES cells that Prep1 preferentially binds promoters, as previously shown in the whole embryo trunk [18].

**Fig 1:**
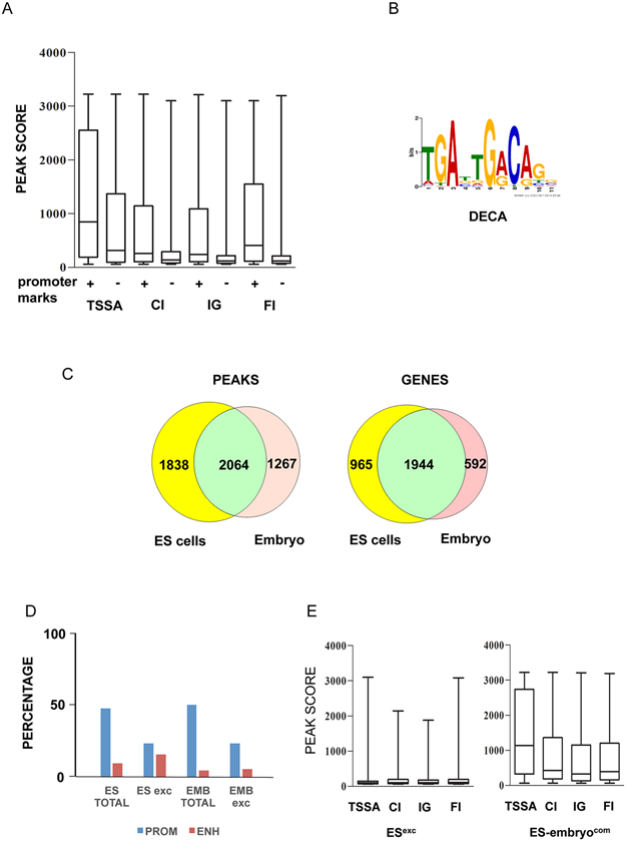
ChIP-seq analysis of Prep1 binding sites in ES cells and comparison to the E11.5 embryo trunk. (A) Subdivision of the Prep1 peaks among peaks -500 to + 100 bp to TSS (TSSA, Transcription Start Site-Associated) with or without promoter marks (H3K4Me3^+^, RNA-Pol-II^+^) (PROM), IG (intragenic), CI (Close-intergenic; less than 20 Kb from a TSS), FI (Far Intergenic; more than 20 Kb from a TSS). We only considered ChIP-seq peaks with p-value <10^-6^ (based on the comparison between immunoprecipitated and non immunoprecipitated input samples). The information on the presence of RNA polymerase II and of the indicated histone modification was taken from [20, 21]. (B) Core Prep1 binding motif (DECA) identified in 70% of all (3902) ES cells peaks. (C) Venn diagrams of ChIP-seq peaks for ES cells and total embryo (left panel) and of the genes corresponding to these peaks (right panel). (D) Promoter and enhancer peaks distribution in ES total, ES^exc^, Embryo total and Embryo^exc^ peaks. Definition of promoters and enhancers as described in the text. The data for the embryo (E11.5 embryo trunks) are taken from [18]. (E) Boxplot and whisker diagrams of the scores of the ES cell-exclusive peaks (ES^exc^) and ES cell-embryo common peaks (ES-embryo com) grouped in TSSA (Transcription Start Site-Associated), IG (Intragenic), CI (Close-Intergenic) and FI (Far Intergenic) regions.

We also observed that the scores (-10[log10 p-value]) of the Prep1 TSS-proximal peaks could be correlated with the presence of the H3K4Me3^+^ and RNA PolII^+^ promoter marks, i.e. that in the TSS-proximal peaks those containing promoter marks were more enriched (Fig 1A).

The DNA consensus sequence targeted by Prep1 (MEME program, ref [23]) was the same observed with the embryo trunk [18], i.e. a decameric (DECA) sequence of the format tGAxtGaCag (Fig 1B), which was present in 2750 (70%) peaks. The Meis1 target sequence tGATtxAT (OCTA) was not enriched among the other targets. However, when we used the Meis1 consensus sequence [18] to interrogate our data set, we found that it could be found with about 10% frequency. As these sequences were observed only in peaks that in the embryo trunk [18] were shared between Prep1 and Meis1, they may represent possible Meis1 binding sites.

Comparison (Table 2) of the Prep1 peaks from ES (this paper) and E11.5 embryo trunks [18] shows that the total number of peaks was not drastically different (3902 v. 3332), but that a major proportion of peaks was ES or embryo-exclusive (ES^exc^ and Embryo^exc^, respectively), with 47.2% among ES v. 38% among embryo peaks (Table 2). When we considered genes rather than peaks, eliminating peaks present more than once per gene, and far intergenic peaks that cannot be assigned with certainty to a specific gene, the numbers decreased but the difference still remained significant (Fig 1C). In ES cells, the number of promoter and enhancers peaks (see above) represented 51% and 10% of the total peaks, respectively, comparable with 54 and 4.7% in the embryo (Table 2). However, among the ES^exc^ peaks, 25% were in promoters and 16.9% in enhancers (25% and 5.8% in the embryo^exc^), which is 3-fold higher than in peaks that are shared with the embryo trunk (Fig 1D).

**Table 2:** Comparison of Prep1 Peaks between ES cells and Embryo Trunk.

	Total Peaks	Promoter Peaks*	Enhancer Peaks^#^
**ES cells total**	3902	2010 (51.5%)	395 (10.1%)
**Embryo total** ^**§**^	3331	1803 (54.1%)	156 (4.7%)
**ES** ^**exc**^	1838 (47.1%)	461 (25%)	310 (16.9%)
**Embryo** ^**exc**^	1265 (38.0%)	317 (25%)	74 (5.8%)

* RNA-PolII^+^, H3K4Me3^+^ See definitions in the legend to Table 1.

^#^ H3K4Me1^+^, H3K4Me3^-^

^§^ from ref [18].

We next compared the median score in the ES^exc^ v. ES-embryo common peaks. As shown in the Boxplot of Fig 1E, the median score in ES^exc^ peaks was clearly lower than in those peaks common to ES cells and embryos, independently of their proximity to a TSS. Thus the ES^exc^ peaks might represent either lower affinity binding sites, or sites that are bound in a smaller sub-population of cells.

Some of the peaks bound by Prep1 only in the ES cells and not in the embryo trunk (ES^exc^) were validated by conventional ChIP analysis using *Prep1* ^-/-^ ES cells and nonimmune IgG as negative controls (S1A Fig). The degree of enrichment obtained by the immunoprecipitation was determined directly by conventional ChIP on the *Suv420h2* gene that is bound in both embryo trunk and ES cells (S1B Fig).

In conclusion, in ES cells Prep1 binds to the same consensus sequence identified in the whole embryo trunk [18]. However a novel set of ES-cells specific genes has been identified, in which the enhancer regions reach >60% of promoters.

### Differential gene expression in WT v. *Prep1* KO ES cells

We have analyzed the transcriptome of wild type and *Prep1* ^-/-^ ES cells (KO2 line) by RNA-seq. 21,446 RNA transcripts from the sense strand were detected in wild type ES cells (S2 Table). From these we considered only those (13,298) RNAs whose level of expression (number of reads per kilobase per million mapped reads, RPKM) was >1, in order to avoid false differential expression caused by oscillations around a too low basal expression. In this group we set an arbitrary threshold to define Prep1-regulated genes, i.e. those in which the effect of the absence of *Prep1* resulted in an over 30% decrease (KO/WT ratio <0.7) or 50% increase (KO/WT ratio >1.5) in expression.

**Fig 2:**
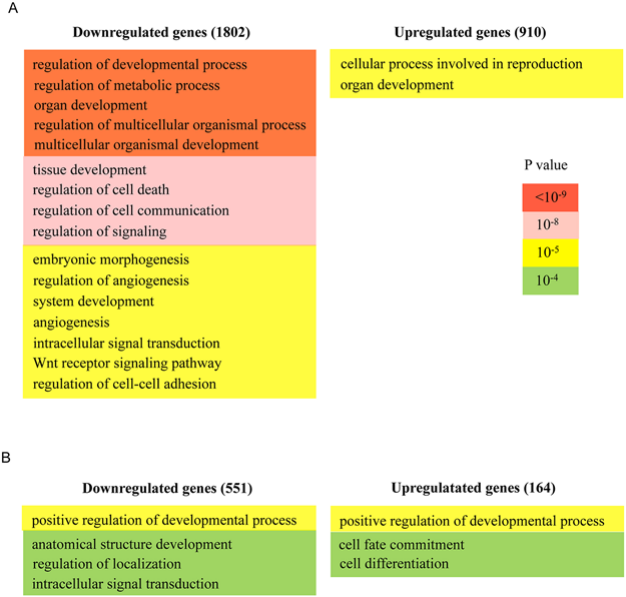
RNA-seq analysis of *Prep1* ^-/-^ ES cells highlights gene categories not enriched in the total embryo. The Gene Ontology terms which are enriched in the *Prep1* ^-/-^ ES cells are shown in (A) whereas the terms enriched only in the fraction of genes bound by Prep1 is shown in (B). The numbers in brackets refer to the number of genes considered. The color code for the *p*-value is shown. Genes are divided in two groups: down- and up-regulated.

We found 910 overexpressed genes in which the *Prep1* ^-/-^/WT RPKM expression ratio was >1.5 and 1802 down-regulated genes in which the ratio was <0.7 (S3 and S4 Tables). Thus, the most frequent effect of the absence of Prep1 was a down-regulation of gene expression. Among the up-regulated genes (S3 Table), GO analysis showed only two statistically significant categories (Fig 2A), one of which being “organ development”. On the contrary, the genes down-regulated in *Prep1* ^-/-^ ES cells (S4 Table) were more significantly enriched in developmental categories like “multicellular organism development”, “tissue development”, “embryonic morphogenesis”, in addition to “regulation of metabolic processes”, “regulation of signaling”, “regulation of cell death” (Fig 2A). It is important to notice that in the RNA-seq analysis of the *Prep1* ^*i/i*^ hypomorphic embryo trunks the “developmental categories” were absent among those significantly enriched by the down-regulation of the gene [18].

In the list of the genes down-regulated in the *Prep1* ^-/-^ ES cells (S4 Table), several enriched genes belong to signaling pathways highly important in embryo development, like Wnt and Fgf. Table 3 and Table 4 show the RNA-seq-based expression of *Wnt* and *Fgf* genes in the *Prep1* ^-/-^ ES cells.

**Table 3:** Effect of the Prep1 KO on the expression of mouse Wnt genes in ES cells#.

gene name	N of total RPKM in WT	KO/WT expression ratio	Adj p-value	corresponding ChIPseq peak
Wnt1	0.036628	1.863555	1*	.
Wnt2	0.086826	**0.223627**	0.2335*	.
Wnt2b	1.085866	0.879216	0.508801*	.
Wnt3	6.423259	**0.275704**	3.29E-52	TSS-proximal (peak 656)
Wnt3a	0.692089	0.549258	0.022601	CI (peak 506)
Wnt4	17.25629	0.56175	2.01E-16	.
Wnt5a	0.528956	0.842651	0.542681*	.
Wnt5b	3.307302	0.582152	2.87E-13	.
Wnt6	5.20061	**0.294619**	1.06E-26	.
Wnt7a	6.1142	0.559066	6.91E-16	.
Wnt7b	19.48256	0.636881	1.95E-31	.
Wnt8a	4.071276	**0.37078**	1.58E-12	.
Wnt8b	0.018909	5.590664	0.132619*	.
Wnt9a	5.154606	0.664204	1.36E-14	CI (peak 506)
Wnt9b	0.157094	**0.254121**	0.003179	.
Wnt10a	2.071242	**0.322174**	2.17E-09	.
Wnt10b	1.043259	**0.474824**	0.001134	.
Wnt11	0.850921	0.602072	0.025877	.
Wnt16	0.295124	0.559066	0.297046*	.

* Statistically not significant.

° RPKM. Reads per kilobase per million.

^#^ The numbers in bold are statistically significant differential expression.

**Table 4:** Effect of the Prep1 KO on the expression of FGF pathway genes in mouse ES cells by RNA-seq#.

gene name	N of total RPKM in WT	KO/WT expression ratio	Adj p-value	corresponding ChIPseq peak
Fgf1	0.109047	**10.11402**	2.32E-40	IG (peak 1691)
Fgf4	50.74752	1.22124	4.97E-22	TSS-proximal (peak 3422)
Fgf5	0.596143	**0.323016**	0.000310539	.
Fgf8	6.561503	**0.250818**	5.32E-47	CI (peak 1845)
Fgf10	0.276518	**0.384358**	0.013002722	.
Fgf13	3.484944	0.76714	0.000413341	.
Fgf17	22.05623	1.538646	4.86E-25	.
Fgf18	1.768287	**3.041802**	2.87E-21	.
Fgf20	0.109687	0.559066	0.651579885	.
Fgf21	2.772174	0.590126	0.025188405	CI (peak 3281)
Fgfr1	73.36409	**0.707726**	4.26E-195	.
Fgfr2	2.836367	**1.582454**	1.63E-21	IG (peak 3393)
Fgfr3	0.671883	1.355312	0.026973184	.
Fgfr4	2.164659	**0.56521**	2.18E-05	IG (peak 934)
Gata4	4.610724	**0.339209**	2.63E-80	.

^#^ The numbers in bold are statistically significant differential expression.

Of the direct target genes bound by Prep1 in their promoters (TSS-proximal), or transcriptional unit (intragenic) or close intergenic regions (Fig 2B) we found 551 genes (about one fourth) in which the *Prep1* ^-/-^/WT RPKM expression ratio was <0.7, and 164 in which the ratio was >1.5 (S3 and S4 Tables). The GO analysis of these genes showed in both groups an enrichment in developmental categories, but down-regulated genes were enriched in “intracellular signal transduction” (Fig 2B).

### Validation of the RNA-seq data in differentiating *Prep1* ^-/-^ ES cells

**Fig 3:**
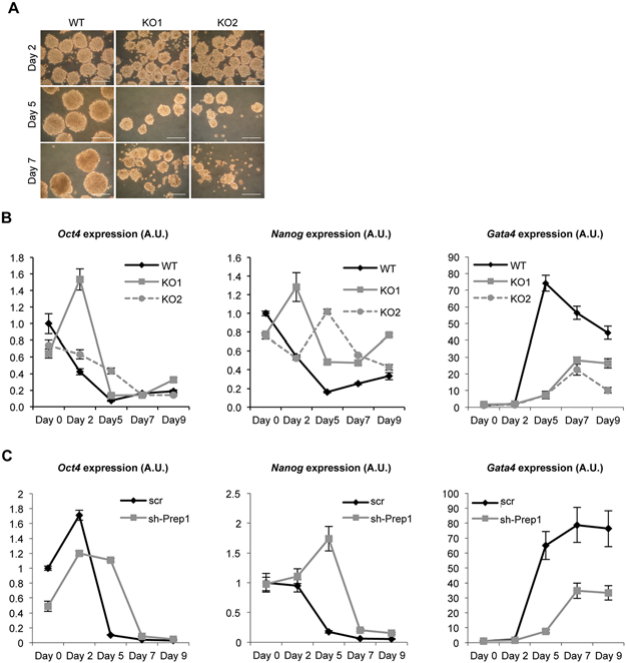
Growth and differentiation are altered in differentiating *Prep1* ^-/-^ embryoid bodies (EBs). (A) Pictures of WT, Prep1-KO1 and Prep1-KO2 embryoid bodies after 2, 5 and 7 days of differentiation. The scale bars indicate 200μm. The EBs were formed starting from 200 cells. (B) RT-qPCR analysis of *Oct4*, *Nanog* and *Gata4* expression in WT, Prep1-KO1 and Prep1-KO2 ES cells (day 0) and in EBs at day 2, day 5, day 7 and day 9 of differentiation (see Methods section). (C) RT-qPCR analysis of *Oct4*, *Nanog* and *Gata4* expression in WT ES cells infected either with a vector containing a scrambled sequence (scr) or with an shRNA targeting *Prep1*. Measurements were carried out at day 0, day 2, day 5, day 7 and day 9 of differentiation (see Methods section).

In order to probe into the physiological significance of the above data we have analyzed the differentiation of independent *Prep1* ^-/-^ ES cell lines *in vitro* (KO1 and KO2). First, we looked at the efficiency of embryoid bodies (EB) formation [19]. *Prep1* ^-/-^ ES cells were previously shown to form EBs, which were smaller than wild-type and displayed increased apoptosis [9]. The smaller size of *Prep1* ^-/-^ EBs was particularly evident at a low (200) ES cell plating number; the difference became progressively more evident during differentiation (Fig 3A). In addition, the external layer of the Prep1 KO EBs, which represents the *in vitro* counterpart of the primitive endoderm in the pre-implantation embryos, was rough and cells seemed to be loose. On the contrary the surface of WT EBs was smooth and cells were tightly attached to each other. Quantitative RT-PCR (RT-qPCR) revealed that during differentiation of Prep1 KO EBs, the stemness markers *Oct4* and in particular *Nanog* were down-regulated later than in WT (Fig 3B). In addition, we found that the induction of the endodermal marker *Gata4* was significantly decreased. Similar results were observed in WT ES cells down-regulated with a *Prep1* shRNA (Fig 3C).

**Fig 4:**
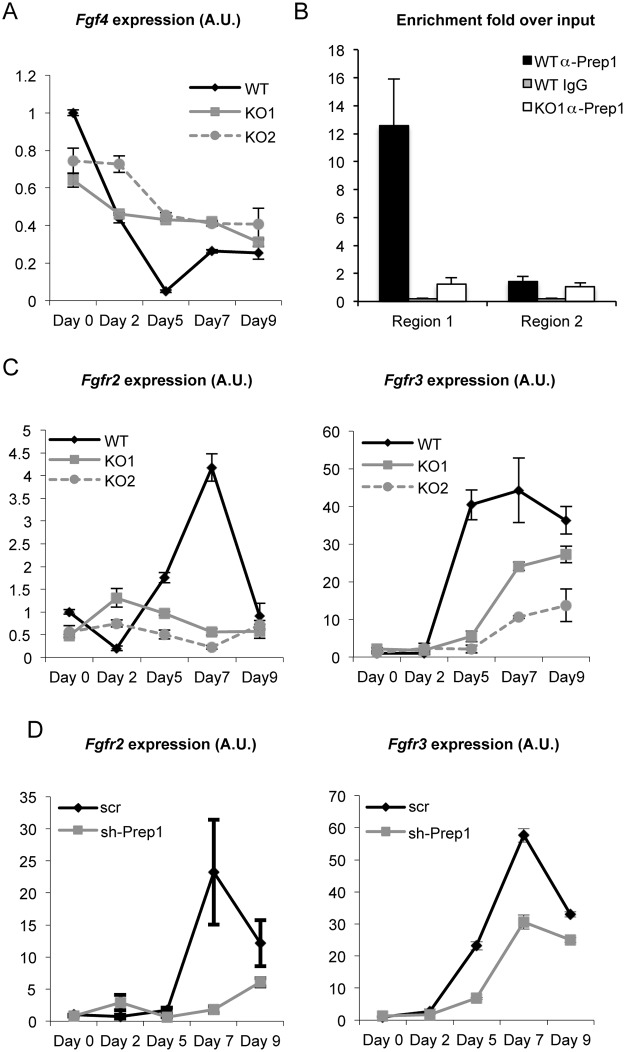
Altered expression of the Fgf pathway during differentiation of *Prep1* ^-/-^ Embryoid bodies (EBs). (A) RT-qPCR analysis of *Fgf4* expression in WT, Prep1-KO1 and Prep1-KO2 ES cells (day 0) and differentiating EBs (days 2–9). Measurements were carried out at day 0, day 2, day 5, day 7 and day 9 of differentiation. Each graph is representative of a total of two experiments in triplicate with essentially identical results (see Methods section). (B) ChIP analysis of the *Fgf4* promoter. Region1 contains the Prep1 binding site, while region 2 does not (see S2 Fig for details on the sequence). The fold enrichment over input obtained by immunoprecipitation with an anti-Prep1 antibody in WT ES cells is shown for region 1 and region 2. As negative controls we used either IgG with WT ES cells chromatin, or anti-Prep1 antibody in the *Prep1* ^-/-^ KO1 ES cells chromatin (see Methods section). (C) RT-qPCR analysis of *Fgfr2* and *Fgfr3* expression in WT, Prep1-KO1 and Prep1-KO2 ES cells (day 0) and EBs at day 2, day 5, day 7 and day 9 of differentiation. Each graph is representative of a total of at least two experiments in triplicate with essentially identical results. (D) RT-qPCR analysis of *Fgfr2* and *Fgfr3* expression in WT ES cells infected either with a vector containing a scrambled sequence (scr) or an shRNA targeting *Prep1*. Measurements were carried out at day 0, day 2, day 5, day 7 and day 9 of differentiation (see Methods section).

RNA-seq data showed that genes of the Fgf pathway are affected by *Prep1* deletion (Table 4) and ChIP-seq analysis included *Fgf4* among the direct targets of Prep1 (S1 Table). This result was of particular interest since Fgf signaling is essential for the first steps of ES differentiation and primitive endoderm formation [24, 25]. In fact RT-qPCR analysis of differentiating Prep1 KO EBs showed an anomalous behavior of *Fgf4* expression. *Fgf4* level was about 30% lower in Prep1 KO ES cells than in WT, but during differentiation it persisted and did not decrease as in WT (Fig 4A). While the mechanism whereby Prep1 regulates the level of *Fgf4* is not known, the direct binding of Prep1 to the *Fgf4* promoter (S1 Table) was also verified by a specific ChIP in ES cells. Indeed, while the genomic region 1 of the Fgf4 promoter (S2 Fig) containing the Prep1 binding site (TGATTGGCAG) was enriched upon immunoprecipitation with an anti-Prep1 antibody, a nearby region 2 lacking this site was not (Fig 4B).

In addition, *FgfR2* and *FgfR3* RNA levels were reduced by two to ten fold in Prep1 KO cells (Fig 4C). Similar results were obtained in WT ES cells down-regulated with Prep1-specific shRNA (Fig 4D). The effect of Prep1 on the *Fgfr*’s must however be indirect, since these genes are not bound by Prep1 in ES cells (S1 Table). A down-regulation of the Fgf pathway in *Prep1* ^-/-^ cells during differentiation might be involved in the delayed formation of the primitive endoderm in *Prep1* ^-/-^ EBs, as shown by a lower and delayed expression of *Gata4* (Fig 3B).

**Fig 5:**
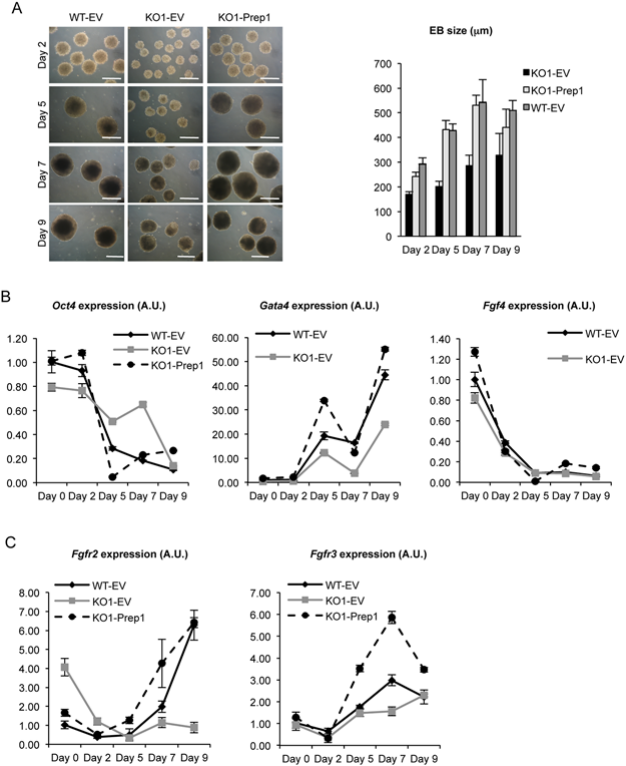
Prep1 reconstitution rescues growth and differentiation of *Prep1* ^-/-^ Embryoid bodies (EBs). (A) Prep1 KO1 ES cells where infected either with an empty vector (EV) or with a vector containing Prep1 cDNA. Their ability to form EBs was assessed along with WT ES cells infected with an empty vector. Pictures of WT-EV, Prep1 KO1-EV and Prep1 KO1-Prep1 EBs after 2, 5, 7 and 9 days of differentiation. The scale bars indicate 200μm. The EBs were formed starting from 1000 cells. The histogram to the right shows the number of EBs formed at different differentiation times in the different cell lines. (B) RT-qPCR analysis of *Oct4*, *Gata4* and *Fgf4* RNA in WT-EV, Prep1 KO1-EV and Prep1 KO1-Prep1 ES cells (day 0) and differentiating EBs at day 2, day 5, day 7 and day 9 of differentiation (see Methods section). (C) RT-qPCR analysis of *Fgfr2* and *Fgfr3* RNA in WT-EV, Prep1 KO1-EV and Prep1 KO1-Prep1 ES cells (day 0) and differentiating EBs at day 2, day 5, day 7 and day 9 of differentiation (see Methods section).

Re-expression of *Prep1* completely rescued the size, appearance and the growth of *Prep1* ^-/-^ EBs (Fig 5A). Moreover, also the levels of *Nanog*, *Gata4* and *Fgf4* were restored and brought back to that of WT (Fig 5B). Indeed, in *Prep1* ^-/-^ cells infected with *Prep1* cDNA, *Nanog* was completely down-regulated during differentiation. Likewise, *Gata4* was induced 40% more after re-expression of *Prep1*. Similarly, *Fgf4* was increased by about 40% and was down-regulated faster during differentiation. Finally, the expression levels of *FgfR2* and *FgfR3* were also rescued (Fig 5C).

**Fig 6:**
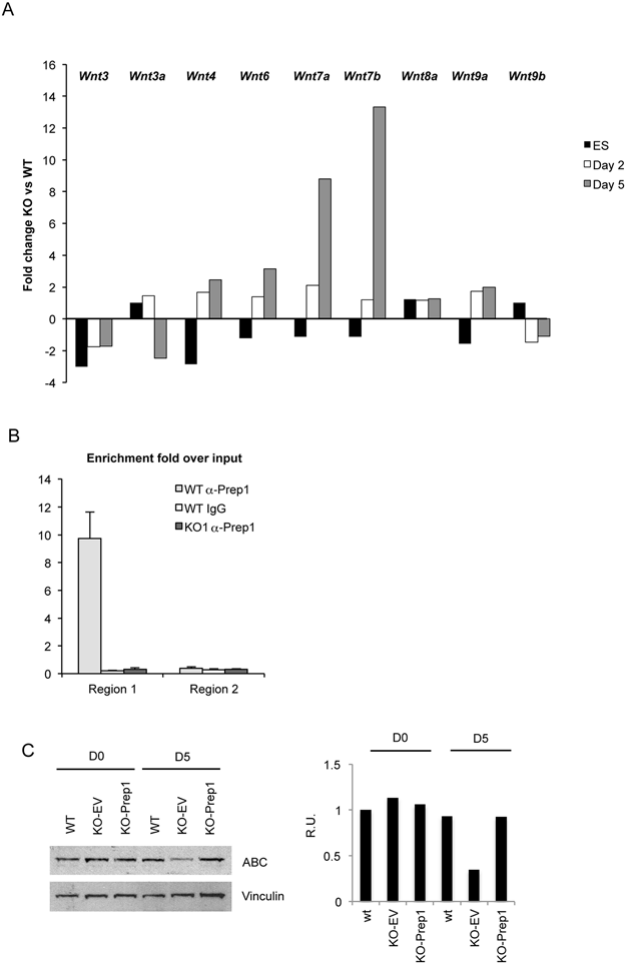
*Prep1* ^-/-^ Embryoid bodies (EBs) express altered levels of various *Wnt* RNAs during differentiation and Prep1 directly targets the *Wnt3* promoter. (A) Deficient regulation of Wnt genes during differentiation of *Prep1* ^-/-^ EB. RT-qPCR analysis of *Wnt* mRNA expression. Fold change variations of *Wnt3*, *Wnt3a*, *Wnt4*, *Wnt6*, *Wnt7a*, *Wnt7b*, *Wnt8a*, *Wnt8b* and *Wnt9b* RNAs in Prep1 WT v. KO1 ES cells at day 0, day 2 and day 5 of differentiation. (B) Chromatin Immunoprecipitation (ChIP) analysis of the Wnt3 promoter. Region 1 contains the Prep1 binding site, while Region 2 does not (see S3 Fig for details on the sequence). The fold enrichment over input obtained by immunoprecipitation with an anti-Prep1 antibody in WT ES cells is shown for region 1 and region 2. As negative controls we used either IgG with WT ES cells chromatin, or anti-Prep1 antibody in the *Prep1* ^-/-^ KO1 ES cells chromatin (see Methods section). (C) Immunobloting of total cell lysates from the WT ES cells (WT), *Prep1* ^-/-^ ES cells transfected with empty vector (KO-EV), Prep1-rescued ES cells (KO-Prep1) before (D0) and after 5 days of differentiation of EB (D5) using an anti-active beta-catenin antibody (anti-ABC). Anti-vinculin antibody was used as loading control. The quantification of the immunobloting is shown in the right panel. Relative units (R.U.) are the band intensities normalized to that of the band of ES WT cells before differentiation.

In agreement with the RNA-seq data (Table 3), we also observed that the time course of *Wnt* genes expression during differentiation of EBs was drastically modified in the absence of *Prep1*. In particular at day 5 of EB differentiation, RT-qPCR data showed that *Wnt3* and *Wnt3a* were less expressed by >60% in *Prep1* ^-/-^ ES cells (Fig 6A). The expression of other *Wnts* was also strongly affected during differentiation of *Prep1* ^-/-^ ES cells (Fig 6A), as also suggested by the RNA-seq data on *Prep1* ^-/-^ ES cells of Table 3. *Wnt9b* was down-regulated by 30%, while others were overexpressed: *Wnt7b* (8-fold), *Wnt7a* (5-fold) and *Wnt6* (2.5-fold). *Wnt8a* and *Wnt9a* showed no change. Thus, since the expression of many *Wnt* genes is affected in the *Prep1* ^-/-^ background, we conclude that Prep1 can also influence the Wnt pathway and its absence may affect Wnt-dependent early embryo development and ES cells differentiation *in vitro*. Indeed, as indicated by the ChIP-seq data (S1 Table) and confirmed by a specific ChIP PCR (Fig 6B), the Wnt3 gene was proven to be a direct target of Prep1. A strong enrichment of the region carrying Prep1 target sequence was obtained by immunoprecipitating chromatin with Prep1 antibody, but not of the nearby region (S3 Fig for the detail on the Wnt3 promoter). IgG control with WT chromatin or anti-Prep1 antibody with *Prep1* ^-/-^ chromatin showed no enrichment (Fig 6B). The reduction of Wnt activity during EB differentiation was confirmed by the decrease of active β-catenin (Fig 6C) at day 5 of differentiation in *Prep1* ^-/-^ cells. The reduction of active β-catenin was rescued by the re-addition of Prep1.

### The absence of Prep1 affects the formation of the Primitive Endoderm *in vivo*

**Fig 7:**
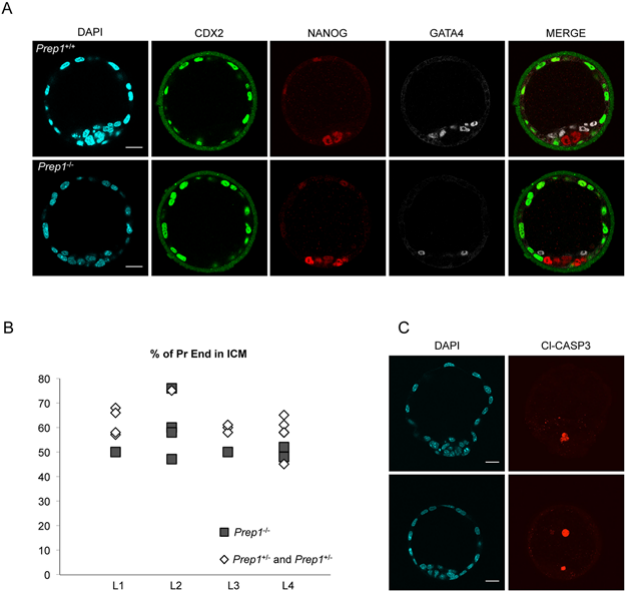
Decreased representation of primitive endoderm (PE) cells in E.4.5 *Prep1* ^-/-^ mouse embryos. (A) An example of confocal sections of immunofluorescent stainings of Cdx2, Nanog and Gata4 in e4.5 *Prep1* ^-/-^ and WT embryos, counterstained with DAPI. The scale bars represent 20μm. (B) Percent of PE cells in the inner cell mass per embryo, at E4.5, in 4 independent litters resulting from *Prep1* ^*+/-*^ intercrosses (L1 to L4). (C) Z projections of confocal sections (covering the whole embryos) showing cleaved Caspase-3 immunostaining in E4.5 *Prep1* ^-/-^ and WT embryos. One confocal section of the DAPI counterstaining is shown for each embryo. The scale bars represent 20μm.

The observation of an altered expression of *Gata4* in Prep1 KO EB prompted us to analyze Prep1 KO embryos at E4.5, which represent their *in vivo* counterpart. *Prep1* ^-/-^ embryos die at about E6.25 due to p53-dependent apoptosis of the epiblast [9]. At E4.5, *Prep1* ^-/-^ blastocysts exhibit a normal morphology, with a well identifiable trophectoderm (Cdx2-positive), Epiblast (Nanog-positive) and primitive endoderm (PE) (Gata4 positive cells) as shown by immunofluorescence (IF) (Fig 7A). However, quantification revealed that among the cells of the inner cell mass, the Gata4-expressing PE cells represented a slightly smaller fraction in *Prep1* ^-/-^ than in WT embryos (Fig 7B), a difference which was statistically significant (*p* = 0.01). This agrees with the reduction of *Gata4* expression in *Prep1* ^-/-^ ES cells and differentiating EBs (Table 2, Fig 3B). At this stage, immunostaining with an anti-cleaved Caspase-3 antibody did not reveal excess apoptosis in *Prep1* ^-/-^ embryos (Fig 7C), indicating that the reduction in Gata4-expressing cells was not due to selective cell death. This represents a novel, precocious phenotype of the *Prep1* ^-/-^ embryos.

### The absence of Prep1 affects the expression of Pbx proteins in ES cells

**Fig 8:**
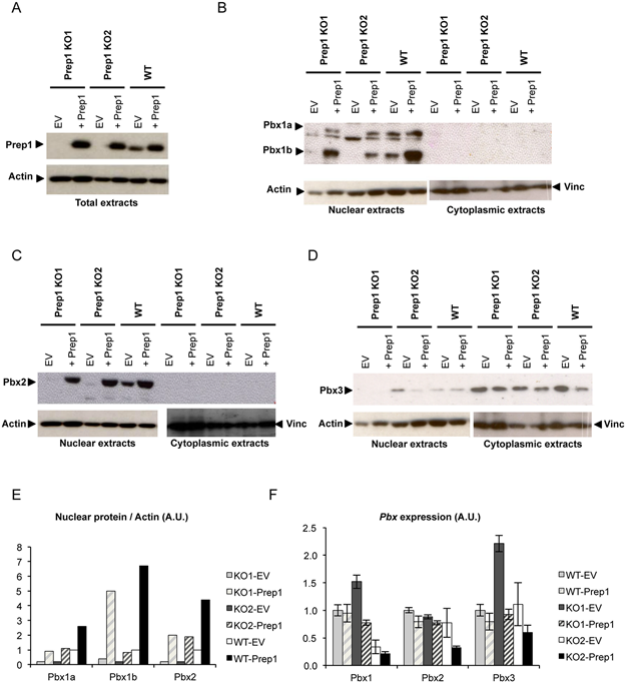
*Prep1* ^-/-^ ES cells express decreased levels of Pbx1 and Pbx2 proteins but not of their RNA. WT as well as Prep1 KO1 and Prep1 KO2 ES cells were infected either with an empty vector or with a vector containing Prep1 cDNA and were subjected to immunoblotting analysis. (A) Immunoblotting analysis of Prep1 expression in total protein extracts of WT, Prep1 KO1 and Prep1 KO2 ES cells infected either with an empty vector or with a vector containing Prep1 cDNA. An Actin immunoblot was used as loading control. (B) Immunoblotting analysis of Pbx1a and Pbx1b expression in nuclear and cytoplasmic protein extracts of WT, Prep1 KO1 and Prep1 KO2 ES cells infected either with an empty vector or with a vector containing Prep1 cDNA. Actin and Vinculin (Vinc) immunoblots were used as loading control of nuclear and cytoplasmic extracts, respectively. Notice the absence of Pbx1 in the cytoplasmic extract. (C) Immunoblotting analysis of Pbx2 expression in nuclear and cytoplasmic protein extracts of WT, Prep1 KO1 and Prep1 KO2 ES cells infected either with an empty vector or with a vector containing Prep1 cDNA. Actin and Vinculin (Vinc) immunoblots were used as loading control of nuclear and cytoplasmic extracts, respectively. Notice the absence of Pbx2 in the cytoplasmic extract. (D) Immunoblotting analysis of Pbx3a expression in nuclear and cytoplasmic protein extracts of WT, Prep1 KO1 and Prep1 KO2 ES cells infected either with an empty vector or with a vector containing Prep1 cDNA. Actin and Vinculin (Vinc) immunoblots were used as loading control of nuclear and cytoplasmic extracts, respectively. Notice the presence of Pbx3 in the cytoplasmic extracts. (E) Quantification of Pbx1a, Pbx1b and Pbx2 immunoblots normalized to Actin. (F) RT-qPCR analysis of *Pbx1*, *Pbx2* and *Pbx3* mRNAs in WT, Prep1 KO1 and Prep1 KO2 ES cells infected either with an empty vector or with a vector containing Prep1 cDNA.

Since Prep1 binds DNA as a dimer with Pbx, and since Pbx1 interacts also with other members of the TALE family, the absence of Prep1 might lead to occupancy of Prep1 sites by other Pbx dimers, i.e. Pbx-Meis1. However, it is known that the absence of Prep1 might also affect the level of its partner Pbx since Prep1 dimerization stabilizes Pbx proteins in some cells [26–28]. The question therefore arises whether the anomalies of the *Prep1* ^-/-^ ES cells reflect only the absence of Prep1 or also the effect of alternative Pbx dimers. If the absence of Prep1 leads to a decrease in Pbx protein level, the latter possibility would be much less likely. We have tested therefore the level of expression of Pbx proteins in *Prep1* ^-/-^ ES cells. We compared one WT to two *Prep1* ^-/-^ ES cell lines (KO1 and KO2) and also infected *Prep1* ^-/-^ ES cells with expression vectors for Prep1 to re-establish its expression. Immunoblotting analysis showed, in addition to the loss of Prep1 in both *Prep1* null ES cell lines (Fig 8A), also a major decrease of Pbx1a, Pbx1b and Pbx2 (Fig 8B, 8C and 8E), whereas no effect was observed for Pbx3 which however was present mostly in the cytoplasm (Fig 8D). Unlike the decrease in protein, *Prep1* null ES cell lines did not show an equivalent decrease in the mRNA of *Pbx1*, *Pbx2* or *Pbx3* (Fig 8F). In *Prep1* ^-/-^ ES cell lines in which Prep1 level had been re-established infecting cells with a *Prep1* expression vector, the level of Pbx1 and Pbx2 was restored (Fig 8A, 8B and 8E). We conclude therefore that the Prep1 deficiency causes a depletion of the Pbx proteins from the nucleus by a post-transcriptional (possibly destabilizing) mechanism, and that the phenotype observed was mostly due to the loss of the Pbx-Prep1 and not to the formation of alternative complexes.

## Discussion

In contrast to its ubiquitous expression [9, 17] Prep1 exerts multiple functions. Indeed, while the ChIP-seq analysis of the E11.5 embryo trunk showed that Prep1 controls mainly basic or house-keeping genes [18], the presence of ES cells-specific Prep1 binding sites reported in this paper supports a direct involvement in the developmental process. Indeed, embryonic phenotypes range from epiblast apoptosis, to brain damage, impaired hematopoiesis and cranio-facial development [9–11, 29, 30].

There are two novel discoveries reported in this paper: the cell type-specificity of at least a fraction of Prep1 binding sites and the direct involvement of Prep1 in the Fgf and Wnt signaling pathways. There is a large difference in binding sites between the ES cells (this paper) and the total embryo trunk [18]. Since the E11.5 embryo represents a mixture of mostly differentiated cells and different types of progenitors, some of the novel ES cells sites may even be specific for stem cells.

The GO analysis of embryo’s DNA-binding sites showed that basic cellular, and not development-related, functions were enriched among Prep1 targets [18]. The GO analysis of the ES cells data, on the other hand, is not enriched in basic cellular functions, but highlights a different set of gene categories, which include signaling pathways involved in embryo development. In particular, the Wnt and Fgf genes are highly enriched among those dysregulated in the *Prep1* ^-/-^ ES cells (Tables 3 and 4). Wnt and Fgf ligands are required for very important processes in embryo development. These were not evidentiated as important Prep1 targets in the total embryo trunk study [18], possibly because of the presence of mixed lineages in the analyzed material.

The RNA-seq expression data have been validated using quantitative RT-PCR and immunoblotting both in ES cells and in differentiating *Prep1* ^-/-^ EBs confirming that the absence of Prep1 has a major effect on the genes of the Fgf (*Fgf4* and two Fgf receptors) and Wnt pathways. At the same time, these studies have validated a role of Prep1 in the ES cells differentiation. Indeed, the level of expression of many genes that are essential for embryo development, as well as the timing of their induction/repression upon ES cells differentiation, was drastically modified upon *Prep1* down-regulation. This is not the first example of TALE proteins control of the Fgf signaling in ES cells, as Pbx1 was already shown to regulate the expression of *Fgf8* [31].

Part of the anomalies observed in the E4.5 *Prep1* ^-/-^ embryo, a minor, but significant, delay of appearance or decrease of PE cells and of their marker *Gata4*, may be correlated with the perturbation of the Fgf pathway. PE formation requires Fgf4 and its receptors and in this process *Nanog* must be down-regulated and *Gata4* up-regulated. Indeed, in *Prep1* ^-/-^ EB the activity of the Fgf pathway is altered, *Nanog* disappearance is delayed and *Gata4* is not up-regulated. These metabolic alterations may also contribute to the subsequent (around E6.0) apoptosis of the *Prep1* ^-/-^ epiblast [9].

The dysregulation of Wnt genes supports previous evidences of a role of Prep1 in Wnt signaling [30, 32]. In addition, it may also hypothetically explain some features of the *Prep1* phenotypes, like the absence of *Hoxb1b* (the zebrafish equivalent of mammalian *Hoxa1*) as well as of *Hoxb2* and *Hoxb4* in the R1–R4 rhombomeres of zebrafish, which has major consequences on the cranial nerves formation and neural crest cells (branchial arch 1) migration and differentiation [29]. Indeed, *Wnt3* and *Wnt3a* are essential for anterior-posterior patterning and their functions are connected with *Hox* genes expression [33, 34].

We have previously observed that some TALE genes can be bound by TALE proteins [18], suggesting possible cross-regulation, although this possibility has not been really explored. Prep1 binds strongly (one of the strongest ChIP-seq peaks) in the vicinity of the TSS of its own gene (*Pknox1*) in both total embryo and ES cells (S1 Table and ref [28]). It would be worthwhile exploring further the actual auto-regulation and cross-regulations of TALE genes.

Another, possibly important, difference between total embryo trunk and ES cells is the three-fold higher binding of Prep1 to enhancers among the ES^exc^ peaks (Table 2). As enhancers are involved in the “regulation” of gene expression more than promoters, this difference might indicate a more subtle regulatory function of Prep1 in ES than in differentiated cells.

Finally, Prep1 mostly binds DNA in complex with Pbx1 at the level of promoters [18]. In *Prep1* ^-/-^ ES cells, the absence of Prep1 significantly affects the level of the Pbx1 and Pbx2 proteins by a post-transcriptional mechanism, leading to a decrease of Pbx proteins. Within the Prep1-Pbx1 dimer, it is Prep1 that drives Pbx1 to bind at a specific DNA sequence [18], while possibly Pbx1 provides the information for the specific (activating or repressing) function [35]. We have not investigated the nature of the Prep1-dependent Pbx1 stabilization. However, a similar effect has been observed in other cells where it was ascribed to inhibition of Pbx1 proteasomal degradation [26–29].

## Materials and Methods

### ChIP-seq

Chromatin immunoprecipitations (IP) were performed using standard methods with anti-Prep1 antibody (N15, Santa Cruz Biotechnology, Santa Cruz, USA). This antibody can also recognize, much more weakly, Prep2 (our unpublished data). Mouse R1 embryonic stem cells [36] (10^8^ cells) were cross-linked in complete medium (10% FBS) containing 1% formaldehyde for 10 min, the reaction was terminated by addition of 125 mM glycine. Fixed cells were washed three times (5 min each) in cold PBS and lysed in LB1 buffer (LB2 buffer containing 0.5% NP-40 and 0.25% triton X-100). Nuclei were then washed in LB2 buffer (10mM Tris-HCl pH = 8 and 200 mM NaCl) to remove detergents and resuspended in LB3 buffer (LB2 buffer containing 0.1% Na-deoxycholate and 0.5% N-lauroylsarcosine). Chromatin was sonicated 5 times for 30 sec at 30% of the maximum power of a Branson 450 sonicator to generate 100–400 bp chromatin fragments. After clearing by centrifugation, sonicated chromatin was incubated with antibody-bound protein A-conjugated magnetic beads (Invitrogen, Carlsbad, USA). For each IP we used 10 μg antibody. IP with rabbit IgG was performed as negative control. After overnight IP at 4°C the bound complexes were washed twice in WB1 (50 mM Hepes-KOH pH 7.5, 140 mM NaCl, 1 mM EDTA, 1% Triton-X100, 0.1% Na-doexycholate), twice in WB2 (50 mM Hepes-KOH pH 7.5, 500 mM NaCl, 1 mM EDTA, 1% Triton-X100, 0.1% Na-doexycholate) and twice in LiCl WB (10 mM Tris-Cl pH 8.0, 250 mM LiCl, 0.5% NP-40, 0.5% Na-deoxycholate, 1 mM EDTA). Immunoprecipitated complexes were eluted from the beads by incubation for 30 min in EB (2% SDS in TE) at 37°C. The eluted material was reverse cross-linked at 65°C overnight and incubated for 1 h at 55°C with proteinase K. The obtained material was extracted with phenol-chloroform and ethanol precipitated. After RNAse treatment, the DNA was purified with a PCR purification kit (Qiagen, Netherlands). About 10 ng of immunoprecipitated DNA were processed for sequencing.

### ChIP-seq data analysis

Chromatin-immunoprecipitated DNA was sequenced using an Illumina GAII analyzer. Single-end 36 bp reads were first mapped with BWA software [37] against the mm9 version of the mouse genome. Peak-calling was performed using MACS peak-calling algorithm [38]. De novo motif discovery was run to identify consensus sequences enriched in the selected regions versus the whole genome using MEME as de novo motif finder algorithm [23]. The set of peak coordinates was intersected with the sets of peaks obtained for total mouse embryo [18] considering as same peaks when overlapping at least by 50%. Individual instances of the core motif within all peaks were searched with a local install of the FIMO (Find Individual Motif Occurrences) program from the MEME suite [23] with default parameters (individual p-value cutoff 10^-4^).

The sequences of the oligonucleotides used for conventional ChIP are listed in S6 Table.

### RNA-seq

For RNA-seq, total RNA was purified from mouse WT and Prep1 KO2 ES cells(see below) [9]. The mRNA was purified and the library for Illumina chromatin sequencing prepared according to the Illumina recommendations. mRNA samples were sequenced using the paired-end 50 bp protocol. Reads (~7M per sample) were mapped and transcript expression estimated using RSEM [39]. This program aligns the reads against a set of predefined transcripts (in our case mouse ensemble 63 gene build) and uses an expectation maximization algorithm to assign reads probabilistically to one of the isoforms of a given gene. The quantification results from RSEM were then analyzed with the Bioconductor package DESeq [40], which fits a negative binomial distribution to estimate technical and biological variability.

### RNA-seq data analysis

Peak overlapping and correlation with RNA-seq data were performed on the Galaxy bioinformatics platform [41, 42]. Peak association with regulated genes was estimated by calculating peak density per megabase in all Ensembl v63 nuclear genes, their promoters and close intergenic regions and comparing with the corresponding values for genes up- or down-regulated in *Prep1* ^-/-^ ES cells. Peak overlap with histone modification data was established by considering 3000 pb around the summit of the histone mark peaks and intersecting with transcription factor peaks.

### ES cells culture and differentiation

*Prep1* WT and two *Prep1* ^-/-^ (KO1 and KO2) C57Bl6 ES cells were derived in the laboratory [9]. They were cultured in 0.2% Gelatin-coated dishes in Glasgow-modified Eagle’s MEM complemented with 15% ES screened FBS (Hyclone, Logan, UT, USA), under standard conditions with 1000 U/ml leukemia inhibitory factor (LIF; Chemicon, CA, USA). Down regulation of *Prep1* in WT ES cells was obtained by infecting the cells with a pLKO.1 vector containing an shRNA against *Prep1* (Thermo Scientific #RMM3981-201784941) or a scrambled sequence for controls (#RHS4080). Overexpression of Prep1 was obtained by infection of the cells with a pMSCVpuro vector containing mouse *Prep1* cDNA while control cells were infected with an empty vector. Embryoid bodies (EBs) were obtained using the standard hanging drops protocol [19], plating two hundred cells per drop in LIF-free ES medium.

### Embryo preparation and immunostaining

All animal experiments were performed in accordance with Institutional Animal Care and Use Committee of IFOM (project #110/11) approved by the Italian Ministry of Health. Animals were kept on a 12/12-h light/dark cycle (lights on at 07:00 h) with free access to food and water. All animal handlings (sacrifice, etc.) were in accordance with the guidelines established by EU (directive 2010/63/EU). Mice were sacrificed by carbon dioxide euthanasia.

E3.5 embryos were flushed according to standard procedures [36] and cultured for 24h in M16 (Sigma) medium at 37°C and 0.5% CO2. E4.5 embryos were then fixed in PFA 4%, permeabilized in PBS/0.25% Triton X-100, blocked in PBS/0.05% Triton X-100/3% BSA and incubated with primary antibodies: mouse anti-Cdx2 (Biocare Medical CM226; 1:100), rabbit anti-Nanog (Cosmo Bio RCAB0002P-F, 1:50), goat anti-Gata4 (Santa Cruz sc-1237; 1:100), rabbit anti-cleaved Caspase-3 (Cell Signaling 9661; 1:200), followed by secondary antibodies: donkey anti-mouse A488-conjugated (Invitrogen; 1/100), donkey anti-rabbit CY3-conjugated (Jackson Labs; 1/400), donkey anti-goat CY5-conjugated (Jackson Labs; 1/400), and DAPI (0.5μg/mL) counterstaining. The images were acquired with a TCS SP2 AOBS confocal microscope (Leica Microsystem) and processed with ImageJ software.

### RT-qPCR experiments

RNA extraction was processed according to the RNeasy (QIAGEN) protocol. After genomic DNA degradation with RNase-Free DNase (QIAGEN), reverse transcription was performed with the Superscript II (Invitrogen). For *Oct4*, *Nanog*, *Gata4*, *Fgf4*, *Fgfr2* and *Fgfr3* expression analysis, cDNAs were subjected to qPCR on Roche LightCycler480 (Roche) using the primers listed in S7 Table. To measure *Wnt3*, *Wnt3a*, *Wnt4*, *Wnt6*, *Wnt7a*, *Wnt7b*, *Wnt8a*, *Wnt9a* and *Wnt9b* expression levels, predesigned RealTime ready assays (Roche) (S7 Table) were run on Roche LightCycler480 (Roche). *Pbx1*, *Pbx2* and *Pbx3* RNA expression were assessed with predesigned Taqman Gene Expression Assay (Applied Biosystems) listed in S7 Table on ABI-Prism 7900 HT sequence detection system (Applied Biosystems). Results were normalized by *Gapdh* gene expression.

### Immunoblotting experiments

Antibodies used were anti-Prep1 (Santa Cruz sc-25282; 1:200), anti-Pbx1 (Cell signaling 4342S, 1:1000), anti-Pbx2 (Santa Cruz sc-890;1μg/mL), anti Pbx3 (kind gift of Michael Cleary, used at a 1:200 dilution), anti-Vinculin (Sigma V9131; 1:10000), anti-Actin (Santa Cruz sc-1616; 1/200) and anti-active beta-catenin (Millipore 05–665; 1:2000).

## Supporting Information

S1 Fig (A) ChIP-seq results were validated in single ChIP experiments in mouse ES cells. Endpoint PCR revealed enrichment of Prep1 peak regions in samples immunoprecipitated with anti-Prep1 antibody (αPrep1). (B) Quantitative real-time PCR confirmed the binding of Prep1 to the Suv420h2 promoter region in samples obtained in ChIP experiments in mouse ES cells (αPrep WT) with much higher enrichment then in *Prep1* ^-/-^ ES cells (αPrep KO).
(PDF)

S2 Fig Fgf4 promoter region. DNA sequence of the *Fgf4* promoter region. Region 1 and Region 2 are highlighted in green and red, respectively. The Prep1 binding site identified by ChIP-seq is underlined in bold blue. Prep1 binding consensus sequences which were not found to be bound by Prep1 in ES cells are underlined in thin blue. The transcription start site is indicated by an arrow.
(PDF)

S3 Fig Wnt3 promoter region. DNA sequence of the *Wnt3* promoter region. Region 1 and Region 2 are highlighted in green and red, respectively. The Prep1 binding site identified by ChIP-seq is underlined in bold blue. The transcription start site is indicated by an arrow.
(PDF)

S1 Table ChIPseq analysis of Prep1 in wild type ES cells. (XLS)

S2 Table RNA-seq analysis of wild type and Prep1^-/-^ C57Bl/6 ES cells. (XLSX)

S3 Table A. RNA-seq analysis of genes overexpressed in Prep1 KO ES cells (KO/WT ratio >1.5). B. Genes down-regulated in Prep1^-/-^ ES cells and bound by Prep1 in wt ES cells. (XLSX)

S4 Table A. List of Prep1 target genes overexpressed in Prep1 KO ES cells (KO/WT ratio >1.5). B. Genes upregulated in Prep1^-/-^ ES cells and bound by Prep1. (XLSX)

S5 Table Gene Ontology Analysis of the Genes Down-Regulated in Prep1 KO ES Cells. (XLS)

S6 Table Oligonucleotide Primers employed in conventional ChIP analysis. (DOCX)

S7 Table Primer oligonucleotides for RNA level measurement. (DOCX)
