# Supplementary figures and images for: ChIP-Seq and RNA-Seq Analyses Identify Components of the Wnt and Fgf Signaling Pathways as Prep1 Target Genes in Mouse Embryonic Stem Cells
Source: PLoS One. 2015 Apr 13;10(4):e0122518. doi: 10.1371/journal.pone.0122518 (PMC4395233; doi:10.1371/journal.pone.0122518)

Supporting S1\_Figure

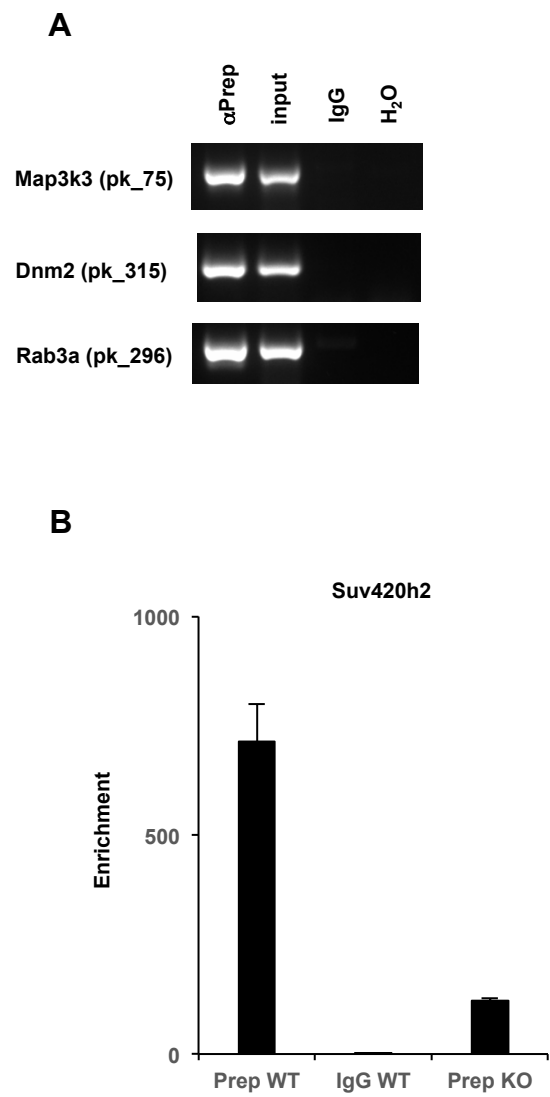

Supplement: S1 Fig — (A) ChIP-seq results were validated in single ChIP experiments in mouse ES cells. Endpoint PCR revealed enrichment of Prep1 peak regions in samples immunoprecipitated with anti-Prep1 antibody (αPrep1). (B) Quantitative real-time PCR confirmed the binding of Prep1 to the Suv420h2 promoter region in samples obtained in ChIP experiments in mouse ES cells (αPrep WT) with much higher enrichment then in Prep1 -/- ES cells (αPrep KO). (PDF) [file pone.0122518.s001.pdf]

Supporting S3\_Figure

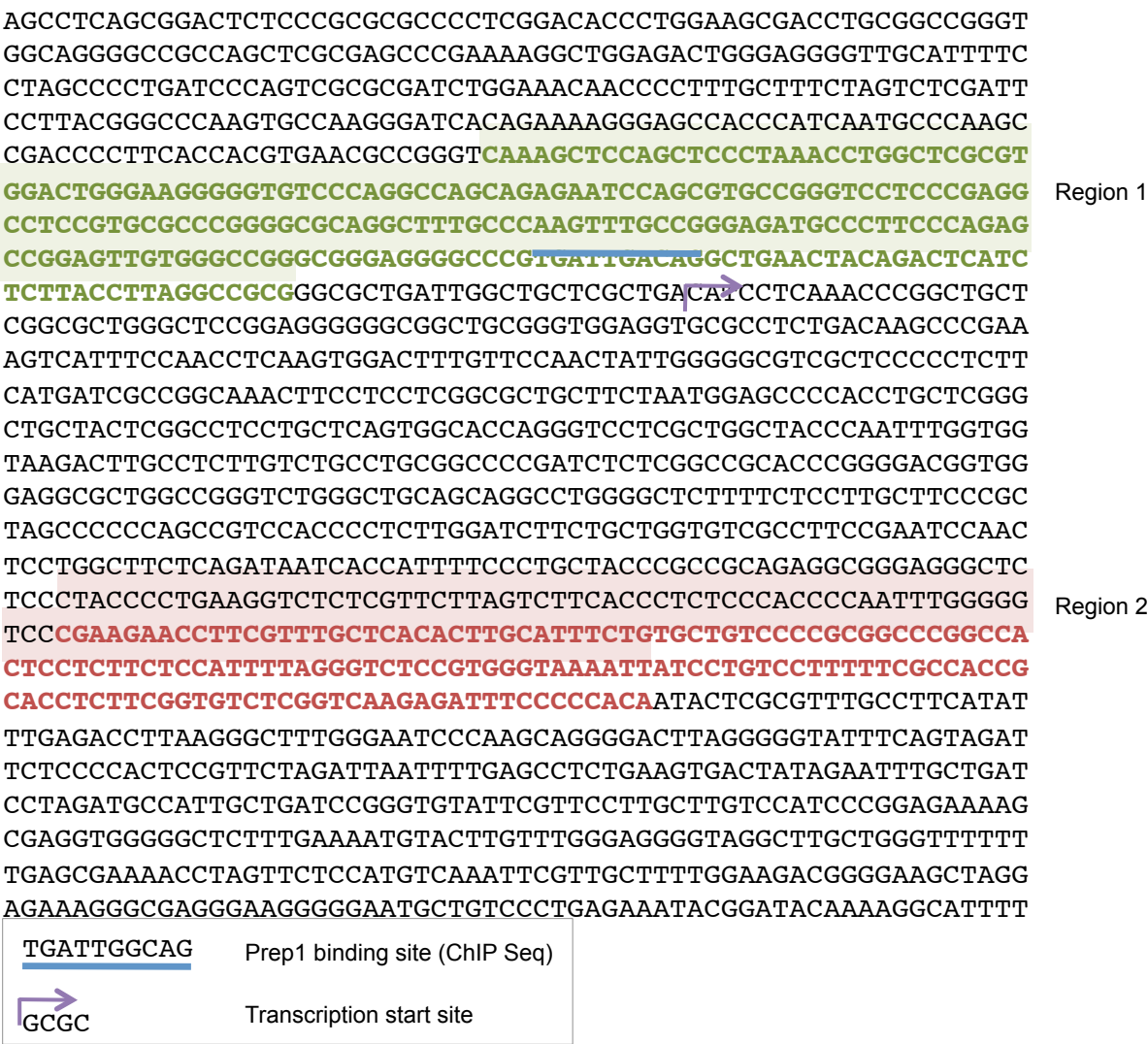

Supplement: S3 Fig — DNA sequence of the Wnt3 promoter region. Region 1 and Region 2 are highlighted in green and red, respectively. The Prep1 binding site identified by ChIP-seq is underlined in bold blue. The transcription start site is indicated by an arrow. (PDF) [file pone.0122518.s003.pdf]
