# Supplementary material for: ChIP-Seq and RNA-Seq Analyses Identify Components of the Wnt and Fgf Signaling Pathways as Prep1 Target Genes in Mouse Embryonic Stem Cells
Source: PLoS One. 2015 Apr 13;10(4):e0122518. doi: 10.1371/journal.pone.0122518 (PMC4395233; doi:10.1371/journal.pone.0122518)
Supplement: S2 Fig — DNA sequence of the Fgf4 promoter region. Region 1 and Region 2 are highlighted in green and red, respectively. The Prep1 binding site identified by ChIP-seq is underlined in bold blue. Prep1 binding consensus sequences which were not found to be bound by Prep1 in ES cells are underlined in thin blue. The transcription start site is indicated by an arrow. (PDF) [file pone.0122518.s002.pdf]

Supporting S2_Figure

```
CTCTAGGTCTCAGGACCTGGCTGCATTTCCGTTGGTGTTCTGAGTAGGACCAGGAATGAT
TGGTGCAAGCCCAATGACAGAGGTTTGGCAGAAGGAAGCCACAGCTCCCAGAAGCTCTGG   Region 2
TCCAAATTACTGATTCCTCGGTGAGGGGGTGCAGCAGGCTCTTCGGCTCCTCCATCAGTG
GCCAGGTCACCTGCTGGCTCCAGGTTCCTCAGATACCACAGAACAGGGACATTCACACCT
AGGAGAACAGTACCAGGGTTCTGCCCAGTTCACCCCCATCAGGCCAGAATGGCCACAGTT
GGGTGTGGGGGTGCTGGTGGGGGAAGGGACCCTTCCAGATCCTTAGAGTTTGACATGCTC
CTGGGTTGGGAAGTTCTGGCCTCCACACTTGAAAATCTCTGGGGAACTGATGCCCAATGT
CTTGCCAGCTTTCTGTCCTTGCCTTGGGTTACCTAGGAGGAAAGTGCGGGTCTCTGCTGT
CCCTGAATGTCCTCAACCTGAGTTCTCTTAGAAGCACCCACTCCGTAGTTTAGACTTCCC
ATCACCTGGAAGACCCTTAAATTTCCAAGCCTCTGGGGCCAGACCAAGCCTTGCCTCCCT
CCCTCCGATCGTGCCCTACCTCCCGGCTCCCTTAGAGTTACACCCTGATAGCCAAGCCAC
AGTGTGACTTGCCTCCCACCGTCAGCTCAAGCCAGTCCTTGGCGCAGCAGAAAGGTTCTG
GCGGTTCACCAAGTGTCCCGCAAGGAAGGAGCACAGGAGATGCCCTGGGGAGCAGAGAGC
CAAGGGGCGGGATCAACAGGTTCGAGTGCGGGGCGGAGCCAAGAGTAAGGGGTTGGGGTC   Region 1
TCTCCAGGTGACAGTAGCCACCGCCAGGCCCGCGCCTCCTCCCCCGGCGGTGATTGGCAG
GCGGCCTGCGCCCCGGCTCCCAGGCGACCGACGCCCCGCGGGGCAGGCGAGTAGGAGGGG
GCGCCGGCTATATATACCACTGCTCCGGAGGGCTGGGCGCGCGGGGACTATCCCGCCACC
GTTGCGTCCCTATTTGCTCTCGCTACTTAGGTCTGTGCGCAGCACTCACCGAACTCACGG
CCCGCAGCTCGAACTCACGCACGGCCCGCGGGCCGGATGGCGAAACGCGGGCCGACCAC
```

TGACAG	Putative Prep1 binding site
TGATTGGCAG	Prep1 binding site (ChIP Seq)
GCGC	Transcription start site
