# Supplementary material for: ChIP-Seq and RNA-Seq Analyses Identify Components of the Wnt and Fgf Signaling Pathways as Prep1 Target Genes in Mouse Embryonic Stem Cells
Source: PLoS One. 2015 Apr 13;10(4):e0122518. doi: 10.1371/journal.pone.0122518 (PMC4395233; doi:10.1371/journal.pone.0122518)
Supplement: S6 Table — (DOCX) [file pone.0122518.s009.docx]

**Table S6**

**Oligonucleotide Primers employed in conventional ChIP analysis.**

| **Gene** | **position** | **sequence** |
| --- | --- | --- |
| Map3K3 | Forward | 5'-TGATTGGCTAGGCGCGAGAG-3' |
| Map3K3 | Reverse | 5'-TCCGAAAAACAAACCGCTGGG |
| Dnm2 | Forward | 5'-CAGGTTGACGTTTCTGCGACC-3' |
| Dnm2 | Reverse | 5'-TGCCACTCAAGAACGTGAGCC-3' |
| Rab3a | Forward | 5'-GTAATCAAGATGTATGCCTGCC-3' |
| Rab3a | Reverse | 5'-CATTGGCTGATGCTCTCGAGC-3' |
| Suv420h2 | Forward | 5'-GGCCTTAACGAATGGAAGCG-3' |
| Suv420h2 | Reverse | 5'-CAGCGAACTACAGCCAATGG-3' |
