# Supplementary material for: ChIP-Seq and RNA-Seq Analyses Identify Components of the Wnt and Fgf Signaling Pathways as Prep1 Target Genes in Mouse Embryonic Stem Cells
Source: PLoS One. 2015 Apr 13;10(4):e0122518. doi: 10.1371/journal.pone.0122518 (PMC4395233; doi:10.1371/journal.pone.0122518)
Supplement: S7 Table — (DOCX) [file pone.0122518.s010.docx]

**Table S7: Primer oligonucleotides for RNA level measurement**

|  |  |  |
| --- | --- | --- |
|  | 5'-3' Forward Primers Sequences | 5'-3' Reverse Primers Sequences |
|  |  |  |
| Gapdh | TCCCACTCTTCCACCTTCGATGC | GGGTCTGGGATGGAAATTGTGAGG |
| Oct4 | GCAGGAGCACGAGTGGAAAGCAAC | TCCCGACTTCCCTTCACCATAC |
| Nanog | AGGCTTTGGAGACAGTGAGGTGC | TACCCTCAAACTCCTGGTCCTTC |
| Gata4 | CTGTGCCAACTGCCAGACTA | CCTGCTGGCGTCTTAGATTT |
| Fgf4 | CCGGTTCTTCGTGGCTATGAG | TTCTTGGTCCGCCCGTTCTTAC |
| Fgfr2 | AAGGTACGAAACCAGCACTGGAG | TCCATCTCCGTCACATTGAACAG |
| Fgfr3 | GCCTGAACCTAGCCAG | CCTTAGCCCAGACCGTG |
|  | | |
| Fgf4-region1 | GGATCAACAGGTTCGAGTGC | AGAGCAAATAGGGACGCAACG |
| Fgf4-region2 | CATTTCCGTTGGTGTTCTGA | TGAATGACCCTGTTCTGTGG |
| Wnt3-region1 | CAAAGCTCCAGCTCCCTAAA | CGCGGCCTAAGGTAAGAGAT |
| Wnt3-region2 | CGAAGAACCTTCGTTTGCTC | TGTGGGGGAAATCTCTTGAC |
|  | | |
| Taqman Gene Expression Assays (Applied Biosystems) | | |
|  |  | |
| Gapdh | ID Mm99999915_g1 | |
| Pbx1 | ID Mm00435507_m1 | |
| Pbx2 | ID Mm00479560_m1 | |
| Pbx3 | ID Mm00479413_m1 | |
|  | | |
| RealTime ready assays (Roche) | | |
|  |  | |
| Gapdh | ID 307884 | |
| Wnt3 | ID 310560 | |
| Wnt3a | ID 301106 | |
| Wnt4 | ID 310523 | |
| Wnt6 | ID 310842 | |
| Wnt7a | ID 310511 | |
| Wnt7b | ID 310567 | |
| Wnt8a | ID 310553 | |
| Wnt9a | ID 310600 | |
| Wnt9b | ID 310838 | |
